# Influence of Postoperative Changes in Sarcopenia on Long-Term Survival in Non-Metastatic Colorectal Cancer Patients

**DOI:** 10.3390/cancers13102410

**Published:** 2021-05-17

**Authors:** Chungyeop Lee, In-Ja Park, Kyung-Won Kim, Yongbin Shin, Seok-Byung Lim, Chan-Wook Kim, Yong-Sik Yoon, Jong-Lyul Lee, Chang-Sik Yu, Jin-Cheon Kim

**Affiliations:** 1 Department of Colon and Rectal Surgery, University of Ulsan College of Medicine and Asan Medical Center, Seoul 05505, Korea; lcyups@gmail.com (C.L.); sblim@amc.seoul.kr (S.-B.L.); crscwkim@amc.seoul.kr (C.-W.K.); yoonys@amc.seoul.kr (Y.-S.Y.); iamleejong@amc.seoul.kr (J.-L.L.); csyu@amc.seoul.kr (C.-S.Y.); jckim@amc.seoul.kr (J.-C.K.); 2 Department of Radiology and Research Institute of Radiology, University of Ulsan College of Medicine and Asan Medical Center, Seoul 05505, Korea; medimash@gmail.com (K.-W.K.); i.am.yongbin@gmail.com (Y.S.)

**Keywords:** colorectal cancer, sarcopenia, skeletal muscle index, overall survival, recurrence-free survival, recovery

## Abstract

**Simple Summary:**

The number of colorectal cancer survivors is increasing due to improvements in oncologic outcomes. Therefore, the risks of metachronous cancer and second cancer are also increased, as well as recurrences. The influence of muscle mass measured as sarcopenia on long-term survival has been studied recently in colorectal cancer patients. Sarcopenia is a factor controllable by lifestyle modification and has gained interest more recently. This study showed an influence of changes in sarcopenia on long-term oncologic outcomes in colorectal cancer and suggests a basis for further investigation of body composition factors, including sarcopenia.

**Abstract:**

The effect of perioperative sarcopenic changes on prognosis remains unclear. We conducted a retrospective cohort study with 2333 non-metastatic colorectal cancer patients treated between January 2009 and December 2012 at the Asan Medical Center. The body composition at diagnosis was measured via abdominopelvic computed tomography (CT) using Asan-J software. Patients underwent CT scans preoperatively, as well as at 6 months–1 year and 2–3 years postoperatively. The primary outcome was the association between perioperative sarcopenic changes and survival. According to sarcopenic criteria, 1155 (49.5%), 890 (38.2%), and 893 (38.3%) patients had sarcopenia preoperatively, 6 months–1 year, and 2–3 years postoperatively, respectively. The 5-year overall survival (OS) (95.8% vs. 92.1%, hazard ratio (HR) = 2.234, *p* < 0.001) and 5-year recurrence-free survival (RFS) (93.2% vs. 86.2%, HR = 2.251, *p* < 0.001) rates were significantly lower in patients with preoperative sarcopenia. Both OS and RFS were lower in patients with persistent sarcopenia 2–3 years postoperatively than in those who recovered (OS: 96.2% vs. 90.2%, *p* = 0.001; RFS: 91.1% vs. 83.9%, *p* = 0.002). In multivariate analysis, postoperative sarcopenia was confirmed as an independent factor associated with decreased OS and RFS. Pre- and postoperative sarcopenia and changes in the condition during surveillance were associated with oncological outcomes.

## 1. Introduction

Sarcopenia refers to muscle depletion. Various methods are used to measure muscle mass and strength, with many being proposed for body composition measurement, including assessments at the atomic, molecular, and whole-body levels. Techniques used include bioelectrical impedance analysis, dilution techniques, magnetic resonance imaging, and computed tomography (CT) [1,2]; many centers generally use CT to measure muscle mass. As CT scans are easily utilized with cross-sectional imaging, enabling measurements at the tissue-system level, they are considered highly accurate for the evaluation of levels of fat, fat-free mass, and skeletal muscle [3,4,5]; the psoas muscle is usually employed to measure skeletal muscle status in patients [6,7].

Recently, many studies have provided evidence of the negative impact of sarcopenia in patients with various types of cancers, such as of the lungs, esophagus, and pancreas [7,8,9]. Advances in chemotherapeutic strategies and surgical procedures are key for longer survival in cancer patients. Postoperative health care during or after surveillance has emerged as an important aspect of treatment, and more patients are in need of postoperative care due to the improved survival in patients with cancer [10]; as survival times increase, so do chances for complications and secondary disease.

In colorectal cancer patients, body composition has been reported to be associated with postoperative complications or overall survival (OS) [4,6,11,12,13,14]; low muscular volume was reported to be correlated with postoperative morbidity [4,6,12].

We previously reported that body composition affected the long-term survival of patients with non-metastatic rectal cancer [15]; rectal cancer patients with sarcopenic obesity and a low BMI at diagnosis had a negative association with OS. Although we showed that body composition was associated with OS, it could not reflect postoperative changes in body composition. We, therefore, aimed to evaluate changes in body composition measured by sarcopenia at diagnosis, as well as at 6 months–1 year and 2–3 years postoperatively. We also investigated the changes in body composition and their association with oncological outcomes.

## 2. Materials and Methods

### 2.1. Study Population

We enrolled patients with (y) *p* stage 0-III colorectal cancer who underwent curative resection at Asan Medical Center between January 2009 and December 2012. Cancer staging was based on the most updated version of the American Joint Committee on Cancer (AJCC) manual at the time of surgery.

Patients who underwent radical resection and elective surgery for primary colorectal cancer, as well as those treated via preoperative chemoradiotherapy followed by radical resection, were included.

**Figure 1:**
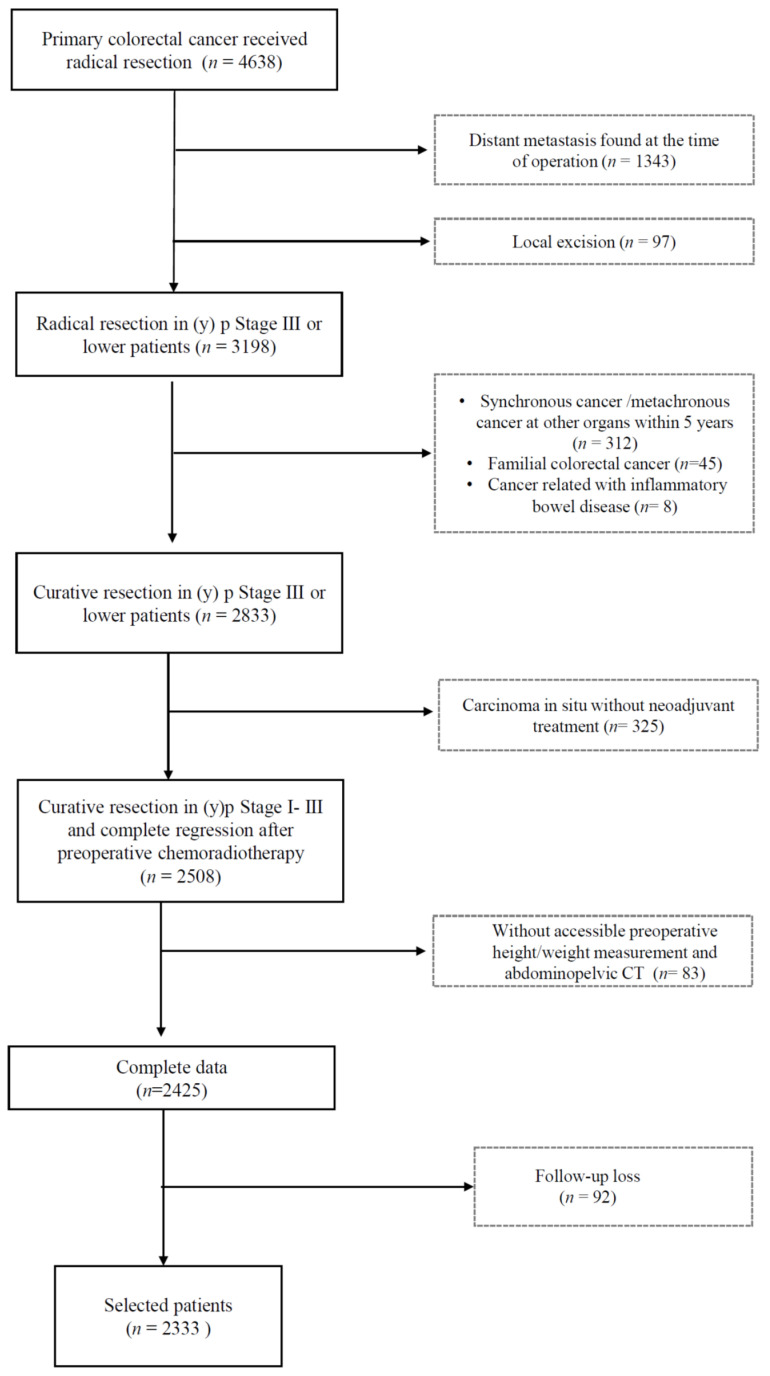
CONSORT diagram.

Patients with synchronous distant metastasis, synchronous cancer at other organs, cancer diagnosed within 5 years, cancer associated with inflammatory disease, who had undergone local excision, or had an unknown staging status were excluded. In addition, we excluded patients with no preoperative height and weight records; those in whom we were unable to calculate skeletal muscle index (SMI) 6 months–1 year and 2–3 years postoperatively; and those who were lost to follow-up observation or did not undergo preoperative and 6 month–1-year and 2–3-year postoperative CT scans. Therefore, 2333 patients who met the inclusion criteria were included in the final analysis (Figure 1).

Data regarding patients’ sex, age, height, weight, pathological stage, and CT images were obtained from medical records; these were used to calculate BMI and body composition.

This study was approved by the institutional review board of the Asan Medical Center, Seoul, Korea (2018–0993). Due to the retrospective design with anonymized data, individual informed consent was waived.

### 2.2. Treatment and Surveillance

Both open and minimally invasive approaches, including laparoscopic or robotic surgery, were performed. Radical resection for rectal cancer was performed according to the principles of tumor-specific mesorectal excision. Adjuvant chemotherapy was initiated 1–2 months after the operation and was recommended for all patients with pathological stage III cancer and those with stage II colon cancer with risk factors such as preoperative obstruction, lymphovascular invasion, perineural invasion, and high tumor budding, and when fewer than 12 lymph nodes were obtained. In patients with pathological stage II-III rectal cancer or those treated with preoperative chemoradiotherapy (PCRT), regardless of pathological stage, adjuvant chemotherapy was recommended.

Recurrences were identified based on imaging modality and confirmed pathologically via biopsy if possible. When pathological confirmation was not possible, a diagnosis was made by combining diagnoses by >2 imaging modalities or serial changes using the same imaging method. Local recurrence was defined as the presence of a suspicious lesion in the pelvis (the site of anastomosis, the bed of the primary resection, etc.) identified using colonoscopy, or imaging modalities such as abdominopelvic CT, magnetic resonance imaging, or positron emission tomography (PET). Distant metastasis was defined as the presence of recurrences beyond the pelvis.

### 2.3. Measurements and Definitions of Body Composition Parameters

All CT scans were retrieved from the Picture Archiving and Communication System (PACS) at Asan Medical Center. The presence of sarcopenia was evaluated according to abdominal CT scans using Asan-J software, developed based on Image J (NIH, Bethesda, MD, USA).

**Figure 2:**
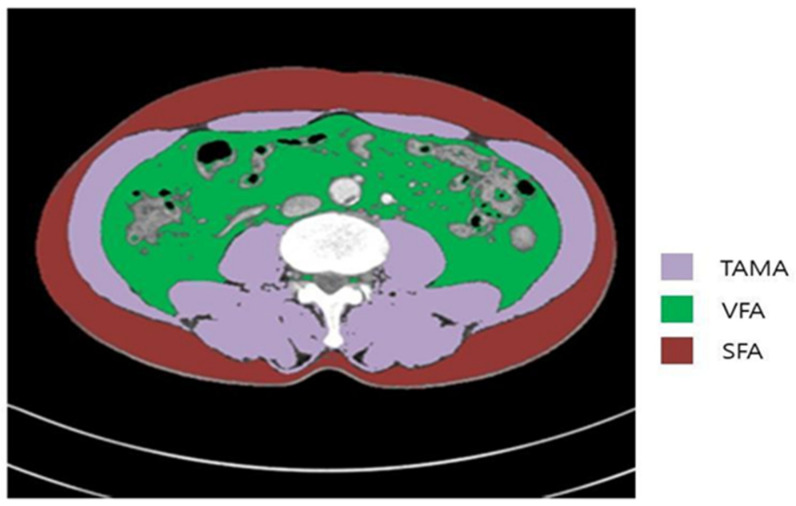
Body morphometric evaluations of abdominal fat and muscle areas. At the level of the inferior endplate of the L3 vertebra, an axial CT image was segmented into the total abdominal muscle area (TAMA), visceral fat area (VFA), and superficial fat area (SFA).

Two consecutive axial CT images at the level of the inferior endplate of the L3 lumbar vertebra were processed and averaged for each patient. The total abdominal muscle area (TAMA, cm^2^)—including the psoas, paraspinal, transversus abdominus, rectus abdominus, quadratus lumborum, and internal and external oblique muscles—was demarcated using predetermined thresholds for the Hounsfield units on CT, or the signal intensity (SI) on precontrast CT. The visceral (VFA, cm^2^) and subcutaneous fat areas (SFA, cm^2^) were also demarcated using the adipose tissue thresholds on CT (Figure 2).

Sarcopenia was defined based on the skeletal muscle index (SMI), which was calculated as TAMA/height^2^. In our previous study, we selected the Western criteria used in a large population-based study; the cut-off values for sarcopenia were an SMI of <38.5 cm^2^/m^2^ in women and <52.4 cm^2^/m^2^ in men [14].

All patients underwent CT scans for the analysis of body composition at diagnosis, as well as 6 months–1 year and 2–3 years postoperatively.

### 2.4. Statistical Analysis

All continuous variables are presented as the mean ± standard deviation (SD). OS was determined from diagnosis until death (all-cause mortality), or the last follow-up date; recurrence-free survival (RFS) was defined as the time from diagnosis until recurrence. Univariate and multivariate survival analyses were conducted using the Cox proportional hazards model to analyze hazard ratios (HRs), from which the 95% confidence intervals (CIs) were obtained. Backward stepwise elimination with a threshold of *p* = 0.10 was used to select variables in the final model. Multicollinearities among correlated variables were checked using the variance inflation factor and condition index.

All evaluations were performed using a 2-sided test; *p* < 0.05 was considered statistically significant. All statistical analyses were performed using PASW Statistics 21 (SPSS Inc., Chicago, IL, USA).

## 3. Results

### 3.1. Clinical Characteristics

**Figure 3:**
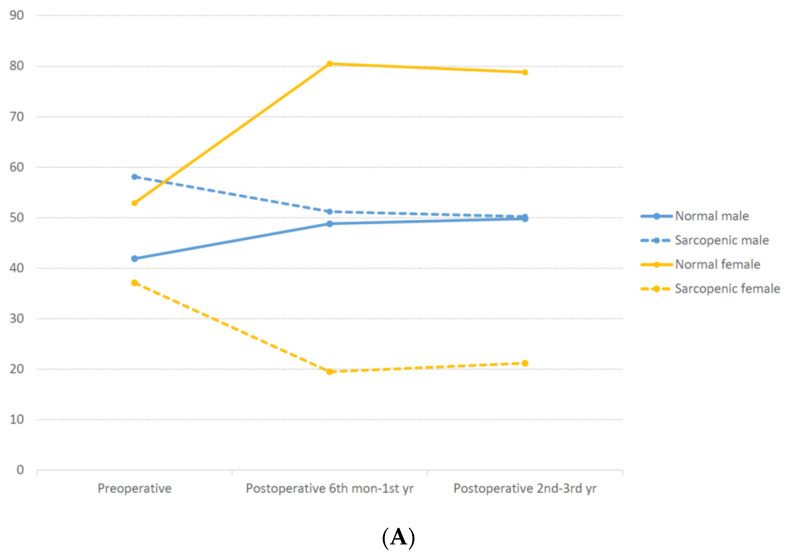

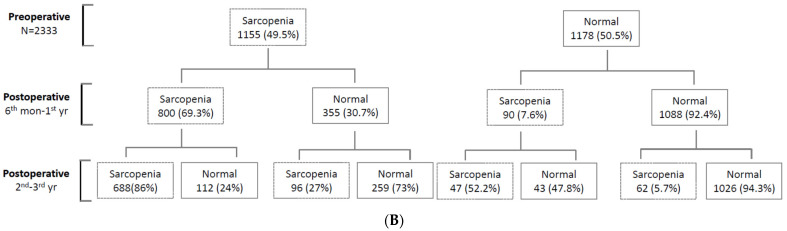
Proportions of sarcopenia and normal body composition during surveillance. (**A**) Changes in sarcopenic status during surveillance. (**B**) Number of patients with sarcopenia or normal status during surveillance according to sex.

The clinical characteristics of the patients are shown in Table 1. A higher percentage of patients had colon (1728, 74.1%), rather than rectal (605, 25.9%) cancer. Among the 605 rectal cancer patients, 261 (43.1%) underwent preoperative chemoradiotherapy. A total of 1387 (59.5%) patients received adjuvant chemotherapy. Among patients who received adjuvant chemotherapy, FOLFOX (35%) was most commonly used, followed by 5-fluorouracil with leucovorin (28.1%), and capecitabine (26.2%). Sphincter-preserving resection was performed on 90.6% of rectal cancer patients. Sarcopenia was preoperatively identified in 1155 (49.5%) patients (male: 799 (58.2%); female: 356 (37.1%)). The proportion of patients with preoperative sarcopenia was similar in females; however, this varied according to age in males, increasing over the age of 40 years (Figure 3).

There were no differences regarding sex, stage distribution, lymphovascular invasion, and perineural invasion between patients with preoperative sarcopenia and normal body composition. Patients with sarcopenia were significantly older (61.7 ± 10.8 vs. 59.1 ± 10.4 years, *p* < *0*.001), and follow-up duration was shorter (60.8 ± 19.1 vs. 62.7 ± 18.3 months, *p* = 0.015); the prevalence of adjuvant chemotherapy was higher in the sarcopenia group (61.6% vs. 57.3%, *p* = 0.037).

### 3.2. Changes in SMI during Surveillance

Among the sarcopenic patients, 67.8% exhibited persistent sarcopenia 2–3 years postoperatively (Figure 3). A significantly higher proportion of patients who recovered normally within 6 months–1 year remained normal 2–3 years postoperatively (24% vs. 72.9%, *p* < 0.001); most patients (90.7%) with a normal preoperative body composition maintained this status 2–3 years postoperatively (Figure 3B).

Changes in the proportion of sarcopenia during surveillance differed between males and females (Figure 3A). Among males, the proportions of sarcopenia and normal body composition decreased over time; however, the proportional difference between sarcopenia and normal body composition was wider 2–3 years postoperatively than preoperatively.

### 3.3. Oncologic Outcomes According to Sarcopenia Status and its Changes

The 5-year OS (95.8% vs. 92.1%, *p* < 0.001) and recurrence-free survival (RFS) rates (93.2% vs. 86.2%, *p* < 0.001) were significantly lower in patients with preoperative sarcopenia. Patients with sarcopenia 2–3 years postoperatively had lower RFS (90.0% vs. 83.2%, *p* < 0.001) and OS (96.3% vs. 90.4%, *p* < 0.001) rates than patients without sarcopenia.

**Figure 4:**
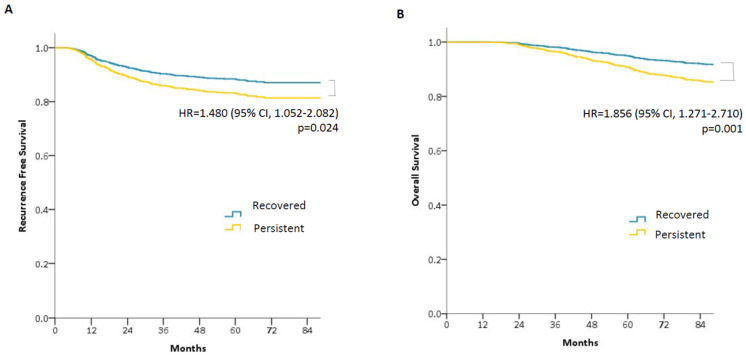
Oncological outcomes according to changes in body composition between preoperative and 2–3-year postoperative status. Recovered to normal body composition (Recovered) vs. persistent sarcopenia (Persistent). Patients with persistent sarcopenia showed both shorter RFS and OS than patients who recovered to normal body composition. (**A**) Recurrence-free survival (RFS); (**B**) Overall survival (OS). HR, hazard ratio; CI, confidence interval.

We further analyzed the oncological outcomes of patients with preoperative sarcopenia according to changes in body composition. The sarcopenia to normal group had a longer OS (*p* = 0.001) and RFS (*p* = 0.002) than the persistent sarcopenia group (Figure 4).

Among female patients, there were no correlations between OS and RFS with changes in the sarcopenia to normal group or in the sarcopenia to sarcopenia group; however, correlations with OS (*p* = 0.013) and RFS (*p* = 0.010) were identified among male patients. 

The sarcopenic status measured at 2–3 years postoperatively was a risk factor of worse RFS in multivariate analysis, contrarily, an increase in skeletal muscle index was associated with longer RFS. Pathological stage, lymphovascular invasion, perineural invasion, and receipt of adjuvant chemotherapy were covariables associated with RFS (Table 2).

Sarcopenia measured at 2–3 years postoperatively showed a negative association RFS and increased skeletal muscle index were positively associated with OS (Table 3); preoperative sarcopenia was a factor related with worse OS Additionally, age, pathological stage, lymphovascular invasion, and perineural invasion were confirmed as risk factors for OS in multivariate analysis.

## 4. Discussion

This study showed that preoperative and 2–3-year postoperative sarcopenic statuses were independent negative prognostic factors for OS, while 2–3-year postoperative sarcopenic status was associated with RFS. Additionally, patients who recovered from sarcopenia to a normal status had a longer OS and RFS than those with persistent sarcopenia.

Sarcopenia has been reported to affect survival in various kinds of malignancies, including colorectal cancer [8,10,13,14,15]; however, criteria and measurement of sarcopenia were inconsistent among studies [13,14,16]. In most previous studies, the associations between sarcopenia and survival were evaluated for a single timepoint [14,15,16]. Although the mechanism through which sarcopenia influences prognosis in patients with cancer is unknown, there is a hypothesis that sarcopenia induces systemic inflammation [2,14], which is also known to increase the risk of cancer and diminish treatment efficacy [17]. The influence of preoperative sarcopenia and inflammatory status on prognosis was studied in a prospective cohort of 2470 patients with stage I-III colorectal cancer [14]; CT scans were used to calculate SMI by measuring muscle status, the same method used in the present study. Over the 6 years of follow-up, they observed that increased neutrophil–lymphocyte ratio and sarcopenia independently predicted OS and cancer-related death. Our previous study also showed that patients with sarcopenia had a significantly lower 5-year OS rate than those without (84% vs. 78%, *p* = 0.003) [15]; however, sarcopenia was not confirmed as an independent risk factor regarding OS in multivariate analyses. Nonetheless, sarcopenic obesity was associated with OS and was more prominent in rectal cancer patients with no inflammatory status. Still, body composition and inflammatory status are liable to change postoperatively; thus, we need to evaluate the effect of changes in body composition on prognosis.

To date, few studies have evaluated the association between postoperative muscle mass changes and oncological outcomes [7,18,19,20]. In both metastatic and non-metastatic colorectal cancer, progressive sarcopenia was reported to be negatively related to survival [19,20]. Our study showed that persistent sarcopenia, as well as sarcopenia at the final evaluation (2–3 years postoperatively), could influence survival. Additionally, quantitative differences between preoperative and 2–3-year postoperative measurements of SMI were also associated with RFS and OS. We, therefore, need to investigate treatment strategies for postoperative sarcopenia.

Changes in sarcopenia status were observed in 20.3% of all patients; in patients with preoperative sarcopenia, 32.1% recovered to normal, while in patients with a normal preoperative composition, only 9.3% of patients experienced newly developed sarcopenia. Based on the results, we determined that preoperative body composition was often sustained. SMI was measured at three timepoints: preoperatively, 6 months–1 year after surgery, and 2–3 years after surgery. Sarcopenia status 6 months–1 year postoperatively would determine body composition 2–3 years postoperatively. Only 13.3% of patients exhibited a change in body composition 2–3 years postoperatively when compared with 6 months–1 year postoperatively. Additional studies are required to determine whether efforts to recover skeletal muscle index early in the postoperative period would influence overall improvement in patients with sarcopenia, resulting in improved survival.

In non-metastatic colorectal cancer, sarcopenia was reported to increase postoperative complications and mortality [6,21,22,23], as well as oncologic outcomes [17,18,19]. A routinely performed preoperative CT scan provides information to quantify sarcopenia and stratify risk groups and give a chance for active nutritional support and risk-stratified postoperative management. In addition, the numbers of patients who receive neo-adjuvant treatment has increased, as 43.1% of rectal cancer patients did in the present study, and we may have a chance of prehabilitation to recover muscle mass to improve surgical and oncological outcomes. One of the main limitations in studies regarding the association between body composition and survival in colorectal cancer, as well as other malignancies, is the lack of a standard definition and consensus regarding the measurement of sarcopenia [13,14,15,16,17,18,19,20]. Abdominopelvic CT is often used to measure skeletal muscle, as it is an essential imaging modality for both preoperative staging and postoperative surveillance; additionally, CT imaging is accepted as a suitable method to measure sarcopenia [2]. Although the European Working Group on Sarcopenia in Older People (EWGSOP) defined sarcopenia as an appendicular SMI of more than two standard deviations below that of healthy adults [2], we used the definition of sarcopenia reported to be related to survival in colon cancer patients in a large cohort [14]. Additionally, the SMI in healthy Korean adults is not well documented; some authors have therefore used the quartile values of the study cohort [13,17].

Asian people were both assumed and reported to have a lower total skeletal muscle mass than Caucasian people; therefore, the proportion of sarcopenia in the present study was expected to be higher than that reported in previous studies [24]. However, patients with preoperative sarcopenia comprised 49.5% of the cohort, similar to the 40.7% reported in studies using the same criteria [14].

Miyamoto et al. [17] used quartile values in Japanese patients, identifying that sarcopenia was significantly associated with higher morbidity in patients undergoing curative resection for colorectal cancer than in patients without sarcopenia. Recently, a large cohort study suggested a standard for sarcopenia in the Korean population in a cohort of 11,845 people [25]. When applying Korean standards to the present study’s cohort, sarcopenia confirmed 2–3 years postoperatively was associated with a poor RFS (HR; 3.90, 95% CI: 2.380–6.391, *p* < 0.001) and OS (HR; 3.902, 95% CI: 2.382–6.392, *p* < 0.001), and was determined to be a risk factor in multivariate analysis; however, the number of sarcopenic patients was very small, accounting for only 4.2% of the total group. It was, therefore, difficult to analyze the difference in oncological results according to changes in sarcopenia before and after surgery; in the future, it will be necessary to analyze the effect of sarcopenia on the prognosis of colorectal cancer in Koreans through a large-scale study applying Korean sarcopenic standards. Considering the various studies that have evaluated the influence of sarcopenia on operative and oncological outcomes in colon cancer patients, sarcopenia would have a negative effect on prognosis. We thus need to come to a consensus, allowing these results to be applied in clinical practice and enabling further studies (including modes of intervention).

In addition to sarcopenia, other types of body composition indicators such as BMI, sarcopenic obesity, and visceral obesity were also studied to evaluate their associations with postoperative and oncological outcomes in CRC or other cancers, as well as with the outcomes of colorectal cancer [26,27,28]. Some studies have reported that sarcopenia was not associated with morbidity or mortality [29,30], while other body composition parameters were found to be related factors.

Considering the various studies that sought to evaluate the influence of body composition on operative and oncologic outcomes in colon cancer patients, abnormal body composition could have a negative effect on prognosis. A consensus regarding the measurement and definition of abnormal body composition is therefore required to apply these results in clinical practice for further study.

In a cross-sectional study of stage I–II colon cancer survivors [30], sarcopenia was not associated with quality of life and was limited to episodes at diagnosis; if patients with sarcopenia are appropriately managed after surgery, the prognosis is good, as reported in our study. As body composition is a modifiable factor, it should be actively managed to improve the long-term outcomes in colorectal cancer patients once the effect of these factors on oncological outcomes has been ascertained.

This study conveyed the limitations that typically underlie retrospective cohort studies. Selection bias meant that we collected patients who had CT scans at the preoperative stage. However, this would not influence inclusion because preoperative CT evaluation is required for all patients who are planning to undergo surgery. In addition, postoperative sarcopenia was evaluated 2–3 years postoperatively; if the follow-up duration were longer, it is unknown whether sarcopenia would have influenced survival in a different way. Lastly, we did not take into account patient medical comorbidities and the related medications that could have affected their body composition.

## 5. Conclusions

In conclusion, postoperative sarcopenia, as well as its changes, affects oncological outcomes. Our results suggest that ‘patient care’ is needed to maintain a positive body composition after surgery and may also affect oncological outcomes; therefore, a large-scale study is needed to verify these results. In addition, it could be possible to further develop this research into a prospective study, to evaluate the role of interventions such as lifestyle modification coaching in oncological outcomes.

## Figures and Tables

**Table 1:** Clinical characteristics of the study patients.

Variables	Mean ± SD, or No (%)
**Age**, years, mean ± SD	60.43 ± 10.70
**Sex**	
Male	1373 (58.8)
Female	960 (41.1)
**Location**	
Colon	1728 (74.1)
Rectum	605 (25.9)
**Pathologic stage**	
Stage (y) 0–II	1546 (66.3)
Stage (y) III	787 (33.7)
**Adjuvant chemotherapy**	
Yes	1387 (59.5)
No	946 (40.6)
**PCRT in rectal cancer patients**	
Yes	261 (43.1)
No	344 (56.9)
**Preoperative Sarcopenia**	
Yes	1155 (49.5)
No	1178 (50.5)
**Surgery**	
Right hemicolectomy	687 (29.5)
Left hemicolectomy	146 (6.3)
Anterior resection	867 (37.2)
Low anterior resection	419 (18.0)
Ultra-low anterior resection	155 (6.6)
Abdominoperineal resection	57 (2.4)
Hartmann’s operation	2 (0.1)
**Number of harvested lymph nodes**	
<12	128 (5.5)
≥12	2205 (94.5)
**Lymphovascular invasion**	
Yes	476 (20.4)
No	1857 (79.6)
**Perineural invasion**	
Yes	380 (16.3)
No	1953 (83.7)

SD, standard deviation.

**Table 2:** Risk factors associated with recurrence-free survival (N = 2333, recurrence = 296).

	Univariate Analysis		Multivariate Analysis	
Variables	Hazard Ratio (95%CI)	*p*-Value	Hazard Ratio (95%CI)	*p*-Value
**Preoperative sarcopenia status**				
Normal	1		1	
Sarcopenia	1.571 (1.245–1.983)	<0.001	1.177 (0.880–1.574)	0.271
**Sarcopenia status at 2–3 years postoperatively**				
Normal	1		1	
Sarcopenia	1.769 (1.408–2.222)	<0.001	1.551 (1.157–2.078)	0.003
**Increase in SMI**	0.213 (0.067–0.678)	0.009	0.246 (0.073–0.827) *	0.023
**Lymphovascular invasion**				
No	1	<0.001	1	<0.001
Yes	2.915 (2.310–3.678)		1.887 (1.304–2.181)	
**Perineural invasion**				
No	1	<0.001	1	<0.001
Yes	3.305 (2.607–4.189)		2.076 (1.614–2.671)	
**Age, years**	0.990 (0.979–1.000)	0.053	0.99(0.985–1.006)	0.364
**Stage**				
Stage (y) 0–II	1		1	
Stage (y) III	2.982 (2.366–3.758)	<0.001	1.618 (1.230–2.219)	0.001
**Sex**				
Male	1		1	
Female	0.732 (0.576–0.931)	0.011	0.875 (0.679–1.126)	0.299
**Adjuvant chemotherapy**				
No	1		1	
Yes	3.501(2.576–4.758)	<0.001	1.911 (1.346–2.713)	0.001

CI, confidence interval; SMI, skeletal muscle index. * Included in multivariate analysis without preoperative sarcopenia status and sarcopenia status at 2–3 years postoperatively.

**Table 3:** Risk factors associated with overall survival (N = 2333, deaths = 237).

	Univariate Analysis		Multivariate Analysis	
Variables	Hazard Ratio (95%CI)	*p*-Value	Hazard Ratio (95%CI)	*p*-Value
**Preoperative sarcopenia status**				
Normal	1		1	
Sarcopenia	2.234 (1.700–2.934)	<0.001	1.447 (1.032–2.027)	0.032
**Sarcopenia status at 2–3 years postoperatively**				
Normal	1		1	
Sarcopenia	2.444 (1.887–3.167)	<0.001	1.806 (1.298–2.513)	<0.001
**Increase in SMI**	0.079 (0.021–0.304)	<0.001	0.110 (0.027–0.449) *	0.002
**Lymphovascular invasion**				
No	1	<0.001	1	0.001
Yes	2.885 (2.225–3.739)		1.653 (1.240–2.204)	
**Perineural invasion**				
No	1	<0.001	1	<0.001
Yes	3.277 (2.516–4.268)		2.176 (1.640–2.887)	
**Age**	1.013 (1.001–1.025)	0.041	1.014 (1.002–1.027)	0.021
**Stage**				
Stage (y) 0–II	1		1	
Stage (y) III	3.200 (2.467–4.151)	<0.001	2.080 (1.514–2.859)	<0.001
**Sex**				
Male	1		1	
Female	0.679 (0.517–0.891)	0.005	0.910 (0.682–1.214)	0.520
**Adjuvant chemotherapy**				
No	1		1	
Yes	2.511 (1.841–3.424)	<0.001	1.282 (0.888–1.850)	0.347

CI, confidence interval; SMI, skeletal muscle index. * Included in multivariate analysis without preoperative sarcopenia status and sarcopenia status at 2–3 years postoperatively.

## Data Availability

Data are available upon reasonable request. We may be able to share de-identified participant data with researchers following the publication of this manuscript. Requests for data should be directed to the corresponding author. Data sharing will need to be approved by third-party data providers.
